# How Does Psychological Distress Due to the COVID-19 Pandemic Impact on Internet Addiction and Instagram Addiction in Emerging Adults?

**DOI:** 10.3390/ijerph182111382

**Published:** 2021-10-29

**Authors:** Giulia Ballarotto, Eleonora Marzilli, Luca Cerniglia, Silvia Cimino, Renata Tambelli

**Affiliations:** 1 Department of Dynamic, Clinical & Health Psychology, Sapienza University of Rome, 00185 Rome, Italy; eleonora.marzilli@uniroma1.it (E.M.); silvia.cimino@uniroma1.it (S.C.); renata.tambelli@uniroma1.it (R.T.); 2 Faculty of Psychology, International Telematic University Uninettuno, 00185 Rome, Italy; l.cerniglia@uninettunouniversity.net

**Keywords:** COVID-19, internet addiction, Instagram addiction, peritraumatic distress, emerging adulthood

## Abstract

International research has underlined a worrying increase in Internet and Instagram addiction among emerging adults during the COVID-19 pandemic. Although the role played by alexithymia and psychological distress due to COVID-19 has been evidenced, no study has explored their complex relationship in predicting emerging adults’ Internet and Instagram addiction. The present study aimed to verify whether peritraumatic distress due to the COVID-19 pandemic mediated the relationship between emerging adults’ alexithymia and their Internet/Instagram addiction, in a sample composed of *n* = 400 Italian emerging adults. Results showed that females had higher peritraumatic distress due to COVID-19 than males, whereas males had higher externally oriented thinking and higher levels of Internet addiction than females. Emerging adults’ psychological distress due to COVID-19 significantly mediated the effect of alexithymia on Internet and Instagram addiction. Our findings supported the presence of a dynamic relationship between individual vulnerabilities and the co-occurrence of other psychological difficulties in predicting emerging adults’ Internet and Instagram addiction during the pandemic, with important clinical implications.

## 1. Introduction

The preventive strategies planned to stem the spread of the virus that causes the Coronavirus Disease 19 (COVID-19) (such as lock-down, home/institutional quarantines, limited mobility, social restrictions, and spatial distancing) [1,2] have resulted in several changes in many aspects of daily life, leading to a substantial increase in Internet use [3,4]. Indeed, the increased social isolation that everyone faced, and the uncertainty of the pandemic situation, led many people to use the Internet to receive information and keep in touch with peers [5,6]. If, on the one hand, the increased Internet accessibility has allowed people to maintain relationships and be able to continue their activities (work, study, etc.), numerous studies have shown a growth in the number of people suffering from Internet addiction [7,8]. Excessive Internet use could disrupt several aspects of life, such as sociality, study or work activities, and physical and psychological health [3]. Youths seem to be the most vulnerable population [3,9,10], with great use of social media, such as Instagram [11].

In particular, research has highlighted the developmental phase of “emerging adulthood” (between the ages of 18 and 25) [12,13] to be a period particularly at risk of the onset of problematic internet use [14,15,16,17] and Internet addiction [18,19]. In fact, emerging adults could use the Internet to undertake the developmental tasks that facilitate a transition to adulthood (such as the achievement of autonomy, the change of relationships with family and peers, the assumption of identity and the intimacy) [20,21]. Epidemiological research on Internet addiction has reported rates of prevalence between 6% and 35% among the young adult population [22,23,24], evidencing the clinical relevance of the phenomenon. Although some studies have found a higher prevalence among males [25,26], more recent evidence has demonstrated increased rates among females, with no sex differences [19,27] or greater rates among females [28,29]. Similarly, while the pre-pandemic literature had shown an increased risk of social media addiction among females [30,31] due to their higher social media use, no differences between males and females in social media addiction were found during the pandemic [32,33].

Given the worrying increase in the number of youths using the Internet in a maladaptive way during the COVID-19 pandemic [7], it is important to identify risk factors associated with this increase to guide preventive programs and reduce the possible long-term consequences on psychological well-being. The Developmental Psychopathology theoretical framework [34] suggests considering the dynamic relationship between the co-occurrence of other psychological difficulties and individual vulnerabilities in studying psychopathological problems among youths (e.g., Internet and/or social media addiction in the time of COVID-19). Consistently, this study aimed to further increase the knowledge on the possible underpinning mechanisms related to Internet/social media addiction in emerging adulthood during the pandemic, exploring the possible role played by emotional difficulties (i.e., alexithymia) and their interplay with psychopathological symptoms resulting from the pandemic.

### 1.1. Psychopathological Risk Due to the COVID-19 Outbreak in Emerging Adults

The COVID-19 outbreak and the related measures put in place to stem the spread of the virus have had a crucial impact on everyday functioning and disrupted personal, social, and work activities worldwide [35,36,37,38,39], especially among emerging adults [40]. Some studies posited emerging adults to be at risk for psychopathological outcomes due to the COVID-19 pandemic and its related restrictions [41]. Indeed, this development phase is characterized by numerous changes (in interpersonal relationships, in the construction of the self and of one’s identity), and the achievement of autonomy is among its main developmental tasks; the restrictions imposed by the COVID-19 pandemic may have made the achievement of these developmental goals more complex [42].

Several studies showed that both isolation and contact restriction among youths can significantly impact their psychological well-being [29,43,44,45,46]. Poor sleep quality, anxiety, distress, depressive symptoms [47], and post-traumatic stress disorder symptomatology [48,49] have been proven to be common responses in different populations during the COVID-19 outbreak, and a higher impact on the female population has also been reported [50,51]. Specifically, in youths, several studies have highlighted that they had higher levels of anxiety, depression, and distress than adults [49,52,53,54]. As regards Italian emerging adults, Parola et al. [55] found an increase in internalizing (anxiety/depression, withdrawal, somatic complaints) and externalizing (aggressive behavior, rule-breaking behavior) problems during the first four weeks of lockdown in Italy, highlighting no differences between males and females. Moreover, national and international studies focused on the psychological impact of the so-called “second wave” of COVID-19 showed increased psychopathological difficulties as the pandemic continues [56,57,58]. 

Interestingly, several studies found significant associations between psychological symptoms due to the COVID-19 pandemic and Internet addiction [59,60,61], as well as between greater emerging adults’ loneliness and increased problematic Internet use [62] and social media use [63]. As also suggested by Cauberghe et al. [64], the increase evidenced in the maladaptive use of the Internet (especially social media) among emerging adults could express a strategy to cope with feelings of anxiety, depression, and loneliness induced by the COVID-19 pandemic and its related restrictions. 

Data from We are social 2021 [65] showed that Instagram was the social media platform that received the largest increase in users in the 18–24 age group in January 2021 (compared to January 2020, before the pandemic). Instagram is a highly visual social media platform that allows sharing one’s own images with other users. The images can be modified with filters to improve the user’s appearance before sharing [66]. With regard to youths’ use, Instagram has been rated as the most potentially harmful among the social media platforms, due to the strong associations with anxious and depressive symptoms [67].

If, on the one hand, before the COVID-19 pandemic, some studies have shown an association between Instagram use and increased psychopathological risk [68,69,70,71], highlighting possible individual vulnerabilities underlying dysfunctional Instagram use [72], the few studies that have evaluated the possible association between emerging adults’ Instagram use and the psychopathological risk during the pandemic have yielded mixed results [5,73]. Specifically, Masciantonio et al. [73] found that active Instagram use was positively associated both with satisfaction with life and with negative affect. Similarly, conflicting results were found by Choi and Choung [5], which highlighted both positive and negative associations between social media and users’ psychological well-being.

Given the lack of studies on this subject and the large increase in the use of Instagram, linked to an upsurge in psychopathological risk, it is considered important to investigate the factors related to the onset of addiction towards this specific social media platform. 

### 1.2. The Role of Alexithymia and Its Complex Interplay with Psychological Distress Due to COVID-19, and Internet and Instagram Addiction

Since before the COVID-19 outbreak, several studies have found alexithymia to be a trait associated with increased psychopathological risk [74,75,76], Internet addiction [77,78], and Instagram addiction [79]. Alexithymia refers to the difficulty in identifying and describing one’s own emotions, with external-oriented thinking and a deficit in emotion regulation [80]. While these characteristics can be considered as the core constructs of alexithymia, several studies have shown that people with high levels of alexithymia exhibit difficulty discerning and evaluating the emotions of others, difficulty in building and maintaining interpersonal relationships, and reduced social skills, etc. [78,81].

Although some studies proposed that alexithymia may be a consequence of psychological distress, such as depression or anxiety [82,83], several other studies agreed that alexithymia is a personality trait [78,84,85,86]. Regarding emerging adults, studies found that male youths have higher levels of alexithymia [87] and specifically externally oriented thinking [88] than females, underlying that youths who show high levels of alexithymia seem to experience more emotional pain and tend to use their virtual interactions to gain social support [75]. In fact, it has been suggested that the Internet may be a tool favored by individuals who have difficulty establishing relationships, due to the absence of the physical presence of others [89]. Thus, individuals who have difficulty in identifying, expressing, and communicating emotions may make excessive use of this tool to better regulate their emotions and meet their unmet social needs. As a result, it can be hypothesized that alexithymia positively predicts the severity of Internet addiction. Based on this hypothesis, and on the fact that the COVID-19 pandemic has led to major changes in interpersonal relationships, we chose to test how the impact of peritraumatic symptoms due to the COVID-19 pandemic may impact the relationship between alexithymia and Internet and Instagram addiction.

Several studies have found an association between alexithymia and mental health problems during the pandemic [90,91,92]. In particular, Tang et al. [93] found a positive association between emerging adults’ mental health problems (such as depression or post-traumatic stress disorder symptomatology) and their levels of alexithymia, during home-quarantine. Moreover, Lin’s study [94] highlighted that the frequent use of virtual social supports and high alexithymia predicted Internet addiction. 

Overall, the international literature has shown that emerging adults’ alexithymia may exert a significant contribution both to Internet addiction [78] and to psychological distress due to COVID-19 [80,90], which in turn plays a key role in predicting Internet addiction [60,61]. This evidence seems to suggest a possible mediating role of peritraumatic distress due to COVID-19 on the relationship between alexithymia and Internet/Instagram addiction. Previous studies have also underlined that individuals’ psychopathological difficulties may mediate the relationship between alexithymic traits and addict behaviors (e.g., mobile phone addiction; [95]), but to date, no study has yet explored these associations in relation to the COVID-19 pandemic. 

### 1.3. The Present Study

During the COVID-19 outbreak, a growing body of research has shown increased psychopathological symptoms due to the pandemic among youths [96,97], and significant associations with alexithymic traits [90,91,92]. In this context, some authors have suggested that youths may tend to overuse the Internet and social media to cope with emotional difficulties, anxiety, depression, and loneliness [64]. Since before the COVID-19 outbreak, several studies have found alexithymia to be a trait associated with higher levels of anxiety, depression, and stress [74,75,76], as well as Internet addiction [77,78] and social media addiction, such as Instagram [79]. However, no study has yet explored the complex interplay between these variables. 

Based on the previous theoretical and empirical premises, the present study aimed to verify the possible mediating role of distress due to the COVID-19 pandemic on the relationship between alexithymia and Internet/Instagram addiction in Italian emerging adults. In particular, this study aimed to: (a) investigate possible differences in the scores of boys and girls on psychological variables; (b) investigate if youths that showed different severities of distress had different levels of alexithymia, Internet addiction, and Instagram addiction, taking into account the role of sex; (c) test the possible mediating role of peritraumatic distress due to the COVID-19 pandemic on the relationship between alexithymia and Internet addiction in emerging adults; and (d) test the possible mediating role of peritraumatic distress associated with the COVID-19 pandemic on the relationship between alexithymia and Instagram addiction in emerging adults.

As regards Internet addiction, studies during the COVID-19 pandemic showed that males manifested increased use of online gaming and online pornography [32,33], with higher levels of Internet addiction. On the other hand, other studies conducted during the pandemic evidenced no sex differences in levels of social media addiction [98,99]. Consequently, we hypothesized that boys showed higher Internet addiction than females, but no sex differences in Instagram addiction. Additionally, in line with Ferrucci et al.’s [97] and Saadeh et al.’s [47] studies, we hypothesized that girls showed greater peritraumatic symptoms due to the COVID-19 pandemic than males. Furthermore, as regards sex differences on levels of alexithymia, based on Kokkonen et al.’s [87] and Chung et al.’s [88] studies, we hypothesized that boys showed higher levels of alexithymia.

Moreover, based on studies that have found significant associations between psychological symptoms due to the COVID-19 pandemic and Internet addiction [59,60,61], social media use [63], and alexithymia [89,90,91], we hypothesized that high levels of alexithymia and of Internet and Instagram addiction were associated with greater severity of psychological symptoms due to the COVID-19 pandemic. 

**Figure 1:**
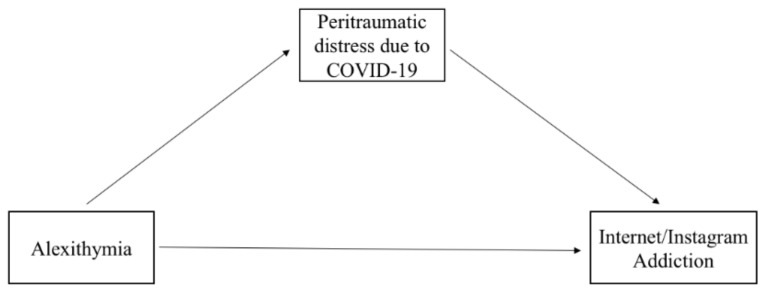
Mediation model to be tested.

Finally, based on studies that highlighted the predictive role of alexithymia on both psychological distress due to the COVID-19 pandemic [90,91] and Internet addiction [78], we hypothesized that peritraumatic distress due to the COVID-19 pandemic mediated the relationship between alexithymia and Internet addiction in emerging adults (see Figure 1). Furthermore, taking into account the contribution of Mei et al. [95], who identified a mediating role of emerging adults’ mental health on the relationship between alexithymia and mobile phone addiction, we hypothesized that the peritraumatic distress due to the COVID-19 pandemic mediated the relationship between alexithymia and Instagram addiction in emerging adults (see Figure 1).

## 2. Materials and Methods

### 2.1. Participants and Procedure

Before the start of this study, the research plan was approved by the Ethical Committee of the Department of Dynamic and Clinical Psychology at Sapienza University of Rome (protocol N. 809/2020), in accordance with the Declaration of Helsinki. 

After obtaining informed consent, each participant filled out self-report questionnaires through an anonymous online survey. Youths filled out some validated questionnaires (described below) and a socio-demographic questionnaire that also explored changes in the use of Internet due to the COVID-19 pandemic. The online survey was spread on social media from 15 November 2020 to 15 March 2021, during the Italian second wave of COVID-19. 

We recruited *n* = 589 Italian emerging adults (age range between 18 and 25 years). From the total sample, we excluded emerging adults who did not complete the assessment procedure (*n* = 106), who reported psychiatric diagnoses and/or physical disorders (*n* = 23 depressive disorders; *n* = 18 anxious disorders; *n* = 19 eating disorders; *n* = 3 borderline personality disorder; *n* = 1 bipolar disorder), and who were following psychological treatment without a specific psychiatric diagnosis (*n* = 17). 

The final sample consisted of *n* = 400 Italian emerging adults (70% females) with an average age of 22.96 (SD = 2.39). Subjects most often reported their highest level of education being high school (41%) or more than high school (56%). The majority (56%) were single, living with their parents (89.7%), in most cases (72.8%) in an intact family, and all youths were Caucasian.

The majority of the sample were students without a job (54.5%). During the COVID-19 pandemic, 73.8% of emerging adults continued to use remote (home) studying, while 10% attended a telematic university since before the pandemic. In total, 2.3 % dropped out of studies because of the pandemic and 8.3% of emerging adults lost their job because of the COVID-19 pandemic.

Further, *n* = 36 emerging adults (9%) tested positive for COVID-19. To verify differences in psychological variables measured between emerging adults infected by COVID-19 and those not infected, a univariate analysis of variance (ANOVA) was carried out. No differences were found (all *p* > 0.05). 

### 2.2. Measures

The COVID Peritraumatic Distress Index (CPDI) [47,100] is a self-report questionnaire composed of 24 items measuring a series of symptoms related to post-traumatic stress disorder (e.g., anxiety, depression, phobias, avoidance behaviors, compulsive behaviors, and loss of social functioning). Items are rated on a 5-point Likert scale ranging from 0 (‘not at all’) to 4 (‘extremely’). The total score ranges from 0 to 100. A score below 28 indicates no distress, between 28 and 51 mild to moderate distress, and above 51 severe distress [47]. Studies have shown a good internal coherence of CPDI [47,100]. In this study, Cronbach α was 0.91.

The Toronto Alexithymia Scale (TAS-20; [101,102,103]) is a 20-item self-report questionnaire assessing alexithymia traits. Each item is rated on a 5-point Likert scale. The questionnaire assessed the three factors theoretically congruent with the alexithymia construct. The first factor (F1) consists of items assessing the ability to identify feelings and to distinguish them from the somatic sensations that accompany emotional arousal. Factor 2 (F2) consists of five items assessing the ability to describe feelings to others. Factor 3 (F3) consists of eight items assessing externally oriented thinking. Higher scores indicate higher alexihtymic traits. The tool demonstrates good psychometrics properties, and it has been found to be stable and replicable across clinical and nonclinical populations [104]. In this study, Cronbach α was 0.98.

The Bergen Instagram Addiction Scale (BIAS; [105]) was developed by adapting the Bergen Social Media Addiction Scale [106,107]. It is a 6-item self-report questionnaire developed to measure six core features of social media addiction: salience, mood modification, tolerance, withdrawal, conflict, and relapse. Each item is scored on a five-point Likert scale from one (very rarely) to five (very often). Higher scores indicate greater Instagram addiction. The tool shows good psychometric properties and, in this study, Cronbach α was 0.86.

The Internet Addiction Test (IAT; [108]) is a 20-item 5-point Likert scale that measures the severity of self-reported compulsive use of the internet. Total IAT scores were calculated, with possible scores ranging from 20 to 100. The scale showed an excellent internal consistency, with a Cronbach α value of 0.91 in this study. According to Italian validation [109], total scores from 0 to 39 represent average users with complete control of their Internet use, scores from 40 to 69 represent excessive Internet use, and scores from 70 to 100 represent significant problems because of Internet use.

### 2.3. Data Analysis

After conducting preliminary statistical analysis (reliability of the measures, frequencies, mean scores, and percentages), and verifying the normality of distribution, an ANOVA was carried out to verify differences in psychological variables measured between emerging adults infected by COVID-19 and not infected. Then, an ANOVA was carried out to verify possible differences between boys and girls on the measured variables. Dependent variables were the CDPI total score, TAS-20 three factors and total score, IAT total score, and BIAS total score. Moreover, a univariate analysis of covariance (ANCOVA) was carried out to verify possible differences on TAS-20 three factors and total score, IAT total score, and BIAS total score, between emerging adults who showed three different levels of distress due to the COVID-19 pandemic (independent variable), considering sex as a covariate. In fact, this analysis was chosen in order to control the possible influence of sex. Finally, in order to verify the presence of a significant correlation between the variables, Pearson’s correlation analyses were carried out. Based on significant correlations and based on the theoretical hypothesis, two mediation analyses were conducted to verify whether emerging adults’ peritraumatic distress due to the COVID-19 pandemic mediated the relationship between their levels of alexithymia and respectively Internet addiction and Instagram addiction. Specifically, the TAS-20 total score was used as an independent variable, the CDPI total score was used as a mediator, the IAT total score and BIAS total score were separately used as dependent variables, and sex was inserted as a covariate. Indirect effects were evaluated with 95% bias-corrected confidence intervals (CIs) based on 10,000 bootstrap samples. All analyses were performed using IBM SPSS software, version 26.0. Mediation analyses were conducted used Hayes’s PROCESS macro [110] (model 4).

## 3. Results

### 3.1. Descriptive Analyses

Descriptive analyses were conducted to observe changes in the psychological variables measured based on demographic variables (see Table 1). 

Furthermore, Table 2 shows descriptive analyses that highlight changes in Internet use from before to after the beginning of the COVID-19 pandemic, both with respect to entertainment and to use for study and/or work.

Table 1 shows the increase in Internet use for both “entertainment” and “for study or work”. On the other hand, data from IAT showed that 88.5% of the sample had complete control of their Internet use, while *n* = 46 emerging adults showed excessive use of the Internet, with symptoms that can be attributed to addiction. 

### 3.2. Assessing Sex Differences

To verify possible differences between boys and girls on measured variables, an ANOVA was carried out. The results are shown in Table 3.

As shown in Table 2, the results highlighted that girls had higher peritraumatic distress due to the COVID-19 pandemic than boys (*p* < 0.001). On the other hand, boys had higher externally oriented thinking and higher levels of Internet addiction than girls.

### 3.3. Assessing Alexithymia and Internet and Instagram Addiction, Based on Different Severities of Peritraumatic Distress Due to the COVID-19 Pandemic

Furthermore, in order to better understand the symptomatology due to the COVID-19 pandemic experienced by emerging adults, descriptive statistics were conducted on CDPI scores. Results showed that *n*= 106 emerging adults (26.5%) showed no distress related to COVID-19, while *n* = 223 (55.8%) showed mild to moderate distress, and *n* = 71 (17.8%) showed severe distress.

To verify if youths that showed different severities of distress had different levels of alexithymia, Internet addiction, and Instagram addiction, an ANCOVA was carried out. The three groups of different levels of distress were considered as independent variables; the TAS-20 three factors and total score, IAT total score, and BIAS total score as dependent variables; and sex as a covariate. The results are shown in Table 4. 

### 3.4. Assessing the Mediating Role of Peritraumatic Distress Due to the COVID-19 Pandemic on the Relationship between Alexithymia and Internet Addiction

We conducted Pearson’s correlation analyses to investigate significant correlations between measured variables (see Table 5).

Based on the results that emerged, to test whether emerging adults’ CDPI total peritraumatic distress due to the COVID-19 pandemic mediated the relationship between their levels of alexithymia and Internet addiction, a mediation analysis was conducted. The TAS-20 total score was used as an independent variable, the CDPI total score was used as a mediator, and the IAT total score was used as a dependent variable. Sex was inserted in the mediation analysis as a covariate.

**Figure 2:**
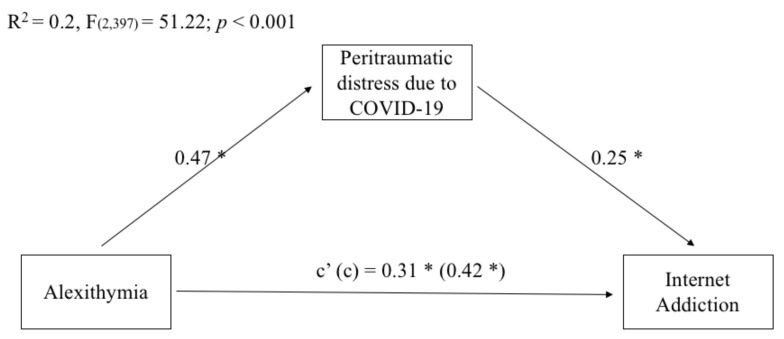
Mediation of emerging adults’ peritraumatic distress due to COVID on the relationship between their levels of alexithymia and Internet addiction. Coefficients shown are standardized path coefficients. c’ = direct effect; c = total effect. * *p* < 0.001.

As can be seen in Figure 2, the results of the mediation analyses showed that the total and direct effects of emerging adults’ alexithymia on Internet addiction were significant. Overall, the model explained 20% of the variance in emerging adults’ Internet addiction. 

Regarding indirect effects, peritraumatic distress due to the COVID-19 pandemic significantly mediated the relationship between alexithymia and Internet addiction (index of moderated mediation = 0.12, BootSE = 0.03, Boot LLCI = 0.07, Boot ULCI = 0.17).

### 3.5. Assessing the Mediating Role of Peritraumatic Distress Due to the COVID-19 Pandemic on the Relationship between Alexithymia and Instagram Addiction

Furthermore, to test whether emerging adults’ peritraumatic distress due to COVID-19 mediated the relationship between their levels of alexithymia and Instagram addiction, another mediation analysis was conducted, using the BIAS total scores as a dependent variable. 

**Figure 3:**
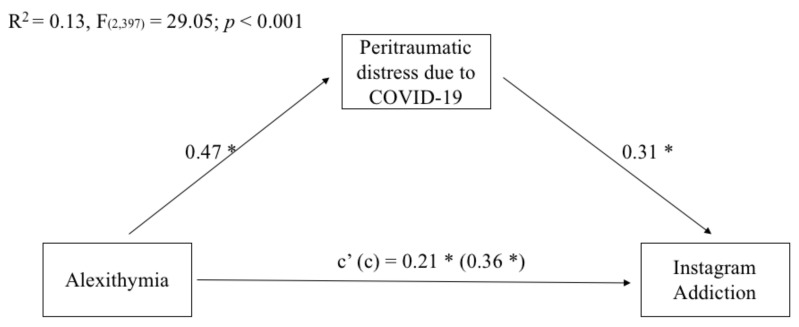
Mediation of emerging adults’ peritraumatic distress due to COVID on the relationship between their levels of alexithymia and Instagram addiction. Coefficients shown are standardized path coefficients. c’ = direct effect; c = total effect. * *p* < 0.001.

As possible to see in Figure 3, the results of the mediation analyses showed that the total and direct effects of emerging adults’ alexithymia on Instagram addiction were significant. Overall, the model explained 13% of the variance in emerging adults’ Instagram addiction. 

Regarding indirect effects, peritraumatic distress due to the COVID-19 pandemic significantly mediated the relationship between alexithymia and Internet addiction (index of moderated mediation = 0.15, BootSE = 0.03, Boot LLCI = 0.09, Boot ULCI = 0.21). 

## 4. Discussion

Since the start of the COVID-19 pandemic, a significant and severe increase in the prevalence of individuals suffering from Internet addiction has been shown [7,8], especially emerging adults [59,111], with important consequences on their psychosocial functioning [3].

Given the clinical relevance of the phenomenon, this study aimed to add new knowledge on the possible risk factors associated with Internet and Instagram addiction among emerging adults during the COVID-19 pandemic. Specifically, we chose to consider the role played by alexithymic traits and psychopathological symptoms based on previous studies that have shown their key contribution in predicting Internet addiction among youths [77,78,79,112,113]. In relation to the specific context of the COVID-19 outbreak, a growing body of studies has also evidenced the risk exerted by alexithymia and psychopathological symptoms specifically resulting from COVID-19 (i.e., peritraumatic distress symptoms) in predicting Internet addiction during the pandemic [60,61,97]. However, no study has yet explored both the direct effect of alexithymia and the indirect effect via emerging adults’ peritraumatic distress due to COVID-19 on Internet addiction and Instagram addiction, and considering the possible role played by emerging adults’ sex. 

Descriptive analyses showed that Internet use for both “entertainment” and “for study or work” has increased. Regarding the prevalence of emerging adults that are Internet addicted, epidemiological studies on Internet addiction during the COVID-19 pandemic have reported highly variable worldwide prevalence rates, from 4.8% to 28.8% [114,115,116], up to 62% [7]. However, to the best of our knowledge, this is the first study that explored the prevalence of Internet addiction during the pandemic among Italian emerging adults, showing that 11.5% of the sample used Internet in an excessive and maladaptive way, indicating a moderate level of Internet addiction in accordance with the IAT cut-off [109].

The first objective was to investigate possible differences between boys and girls on the psychological variables measured. In line with our hypotheses, in the present study, boys showed higher levels of Internet addiction than girls. Research in the field of Internet addiction among emerging adults has shown mixed results, with some studies evidencing higher rates of prevalence among males [113] while other studies have reported no sex differences [117,118] or that females were more likely to be affected by Internet addiction than males [119,120]. However, in line with our findings, the research specifically focused on Internet addiction during the COVID-19 pandemic has also shown a higher risk among males [32]. This could be due to the fact that males showed lower self-control [121] and a greater tendency to engage in high-risk addictive Internet behaviors than females, including gameplay and cybersex activities [122,123]. During the pandemic, this general tendency seems to be also reflected in increased consumption of online pornography, as evidenced by the study of Sallie et al. [32] and Delcea et al. [33], and consequently in higher hours spent online by males than females (a factor commonly related to a higher risk of developing Internet addiction; [124,125]).

On the other hand, in line with our hypotheses, we did not find significant sex differences in Instagram addiction. Pre-pandemic studies on social media addiction have shown a higher risk of Instagram addiction among female youths [30,31], due to their tendency to use the Internet for reasons more related to social feedback and connectedness than males [126,127]. However, studies conducted during the pandemic [91,92] evidenced no significant differences in levels of social media addiction between males and females, suggesting that the social isolation due to the COVID-19-related restrictions has led to a substantial increase in virtual interactions throughout the youth population (beyond possible pre-pandemic gender-related differences) as a strategy for coping with feelings of loneliness and associated psychological distress [64,128].

Moreover, the results on sex differences on peritraumatic distress due to the COVID-19 pandemic confirmed our hypothesis, highlighting that girls had higher peritraumatic distress due to the COVID-19 pandemic than boys. Although the study by Parola et al. [55] found no sex differences on psychopathological symptoms during the first lockdown in Italy, other studies have shown higher psychopathological symptoms due to COVID-19 in females [47,94]. However, unlike these studies, which looked at the psychological impact relative to the first lockdown, the present study assessed COVID-19-related symptomatology more than a year after the first lockdown, so possible differences could be due to the prolonged pandemic. Another possible explanation could be the fact that, as suggested by Bangasser et al. [129,130], female gender represents a biological factor commonly associated with greater psychological vulnerability in the face of stressful life events, resulting in higher peritraumatic symptoms compared to males [131]. In line with our findings and the studies by Bonati et al. [51] and Jiménez et al. [132], this greater vulnerability to stress-related psychopathological sufferance has also been confirmed in relation to symptoms of peritraumatic distress due to COVID-19. 

Again, with regard to sex differences, the findings showed that boys had higher externally oriented thinking than girls. This result is in line with our hypotheses and several studies that found higher levels of alexithymia [131] and specifically of externally oriented thinking in male youths [132]. In this context, in accordance with the normative male alexithymia hypothesis posited by Levant [133,134], and supported by the recent scientific literature [135], males may be inclined to show more alexithymic traits than females due to gender-related social pressure, implying that males should not freely express difficult or attachment feelings. 

As regards emerging adults’ levels of peritraumatic distress due to COVID-19, descriptive statistics highlighted that 55.8% of the sample showed mild to moderate distress, and 17.8% of the sample showed severe distress due to the COVID-19 pandemic. Our results are higher than results found by several other studies on adult populations and the early stage of the COVID-19 pandemic [51,52,136]. Prati and Mancini [137] conducted a meta-analysis of studies investigating the psychological impact of the COVID-19 pandemic, finding great heterogeneity in studies’ results, suggesting that lockdowns have had different effects on mental health. However, our results supported the evidence that distress due to COVID-19 was higher among youths whereas it decreased with age [132], and that individuals’ psychological sufferance has increased as the pandemic has continued [56,57,138]. With regard to our second objective, our findings showed that emerging adults who experienced no distress related to the COVID-19 pandemic showed lower levels of alexithymia, and lower scores on Internet and Instagram addiction. On the other hand, emerging adults who experienced severe distress due to the COVID-19 pandemic showed the greatest difficulties in describing feelings and the highest scores of Internet and Instagram addiction. These findings are in line with our hypotheses and with numerous studies showing alexithymia to be among the individual vulnerabilities underlying a higher psychopathological risk [110,139,140,141], especially resulting from stressful life experiences [142,143]. An emerging adult with difficulties in regulating and identifying his/her emotions may be at higher risk in coping with stressful events, due to a deficit in symbolic thinking [144] and in processing and regulating his/her feelings-related responses [145]. Recently, the predictive effect of alexithymic traits on psychopathological symptoms resulting from the COVID-19 pandemic has also been reported [90,91,146]. Moreover, a significant contribution exerted by emerging adults’ alexithymia in the development of Internet addiction [77,78] and Instagram addiction [79] has also been shown and confirmed during the pandemic too. Moreover, associations that we found between emerging adults’ emotional-behavioral difficulties (i.e., alexhytimic traits and peritraumatic distress) and Internet and Instagram addiction confirmed previous studies in the field [59,62,64] and supported the complex relationship between these variables that we have hypothesized. 

Specifically, the third objective of this study was to test the mediating role of peritraumatic distress due to the COVID-19 pandemic on the relationship between alexithymia and Internet addiction. As hypothesized, results showed that emerging adults’ levels of alexithymia significantly predicted levels of Internet addiction and that peritraumatic distress due to the COVID-19 pandemic mediated this relationship. Moreover, in line with our hypotheses, peritraumatic distress due to the COVID-19 pandemic also mediated the relationship between alexithymia and Instagram addiction. If, on the one hand, the variance explained in the first model was higher than the variance explained in the second model (20% vs. 13%), the peritraumatic distress mediation effect was higher in the second model. This result is specific to emerging adults. Indeed, the literature has indicated that emerging adults represent the population that has experienced the greatest increase in Instagram use since the COVID-19 pandemic began [65]. In accordance with Cauberghe et al. [64], we may hypothesize that emerging adults used Instagram to cope with psychopathological difficulties that emerged during the pandemic; in particular, we speculate that youths who had difficulties in identifying and describing feelings were likely to show psychopathological symptoms and use the Internet (and especially Instagram) to cope with the difficulties they were experiencing. Indeed, several studies have underlined the associations between psychopathological symptoms and Instagram use [147,148], and Ershad and Aghajani [79] found that an underlying vulnerability factor was alexithymia. On the other hand, it should be noted that the variance explained by the model is modest (13%). This can be explained by other factors involved in the development of Instagram addiction, which were not considered in this study. In particular, several studies have shown that a personality trait, such as self-liking [149], or attachment styles [79], or the recognition and social needs’ influence on Instagram addiction [150], have a significant influence on Instagram addiction. Further studies should evaluate the relationships between these factors and their impact in the onset of Internet and Instagram addiction.

This study has some limitations. First, this is a cross-sectional study and we assumed that the associations we found were caused by the COVID-19 outbreak, but our results should be tested by further longitudinal studies. Moreover, the online survey method used could have led to respondence and reporting biases and further studies should confirm our findings by using probability sampling techniques. Furthermore, there was also an overrepresentation of the girls, which could lead to selection bias. Finally, the emerging adults’ family context was not considered. In fact, several studies have highlighted the psychological impact of the family on the origin of the youths’ mental health [105,113,117,151,152].

Despite these limitations, this study has several strengths. This is the first study that tested the role of emerging adults’ peritraumatic distress due to the COVID-19 pandemic in the relationship between alexithymia and Internet and Instagram addiction. Furthermore, our results have several potential clinical implications. For instance, the possibility of identifying emerging adults’ risk factors (such as high levels of alexithymia) could allow the implementation of prevention programs for Internet and Instagram addiction. Furthermore, paying attention to alexithymia and peritraumatic symptoms could allow treatment of the symptoms underlying addict behaviors. Indeed, the psychopathological impact of the COVID-19 pandemic has caused a large increase in psychopathological symptoms and Internet addiction; it is necessary to implement prevention and treatment programs for emerging adults, a population particularly at risk in this period.

## 5. Conclusions

This study evidenced the presence of a dynamic relationship between individual vulnerabilities and the co-occurrence of other psychological difficulties in predicting emerging adults’ Internet and Instagram addiction during the COVID-19 pandemic. In particular, our findings showed that emerging adults’ levels of alexithymia significantly predicted the levels of Internet and Instagram addiction and that peritraumatic distress due to the COVID-19 pandemic mediated this relationship. This result could allow the planning of prevention and intervention programs targeted at helping emerging adults with individual vulnerability (i.e., high levels of alexithymia) to face the current health emergency and preventing peritraumatic distress due to the COVID-19 pandemic and Internet/Instagram addiction. Specifically, treatment strategies focused on supporting youths’ emotion regulation and the ability to recognize and discriminate one’s own and others’ emotions may be more effective in preventing and/or reducing short- and long-term psychopathological consequences related to the COVID-19 pandemic, as well as Internet and Instagram addiction. Moreover, as suggested above, further studies should investigate the role of other variables, such as personality traits or attachment styles, in the complex interplay between alexithymia and Internet/Instagram addiction. Finally, it is important to underline that recent studies are evaluating the presence of peritraumatic symptoms due to the long-COVID syndrome [153,154]. If, on the one hand, in our sample, no differences were found in the psychological variables measured between emerging adults infected by COVID-19 and those not infected, on the other hand, emerging adults infected by COVID-19 only represented 9% of the sample and no data were obtained on the date on which the subjects were infected. Further studies should explore the link between peritraumatic symptoms in emerging adults with and without long-COVID syndrome, to verify possible differences in its mediating effect on the relationship between alexithymia and Internet/Instagram addiction.

## Figures and Tables

**Table 1:** Means and standard deviations of emerging adults’ scores on CDPI, TAS, IAT, and BIAS, based on demographic variables.

	CDPI	TAS-20 Tot	IAT	BIAS
Level of education				
Primary school (*n* = 1)	49	71	3	13
Middle school (*n* = 11)	37.82 (15.48)	58.73 (13.67)	28.64 (15.82)	13.64 (4.63)
High school (*n* = 164)	39.77 (15.86)	54.07 (12.03)	26.02 (13.7)	12.93 (6.08)
College (*n* = 224)	34.6 (13.02)	47.65 (12.55)	21.96(11.79)	11.59 (5.29)
Employment				
Unemployed (*n* = 22)	38.91 (15.54)	54.45 (11.63)	28.14 (9.96)	14 (5.54)
Student (*n* = 218)	38.68 (14.57)	50.83 (13.19)	24.37 (13.24)	12.61 (5.64)
Part-time worker (*n* = 21)	37.86 (16.65)	52.14 (8.97)	22.33 (9.87)	10.67 (4.6)
Full-time worker (*n* = 64)	32.19 (12.33)	50.77 (12.09)	21.2 (11.41)	11.14 (5.16)
Working student (*n* = 75)	34.61 (14.27)	48.47 (13.51)	23.28 (14.28)	11.81 (6.14)
Living with				
Alone (*n* = 17)	25.41 (14.19)	48.76 (10.48)	19.76 (12.44)	11.12 (5.19)
Roommates (*n* = 45)	34.91 (11.89)	47.49 (12.81)	24.36 (10.13)	12.73 (5.22)
Spouse (*n* = 30)	31.7 (14.26)	43.43 (10.73)	20.57 (9.98)	10.27 (5.01)
Parents (*n* = 308)	38.26 (14.53)	51.91 (12.86)	24.20 (13.48)	12.37 (5.75)

Note. CDPI = COVID Peritraumatic Distress Index; TAS-20 Tot = TAS-20 Total score; IAT = Internet Addiction Test; BIAS = Bergen Instagram Addiction Scale.

**Table 2:** Hours per day that emerging adults spent on the Internet for entertainment and study/work activities before and after the beginning of the COVID-19 pandemic.

Hours Per Day	Before the Pandemic	Since the Beginning of the Pandemic
Entertainment		
No use	1	1
<2 h per day	95	41
2–4 h per day	157	102
4–6 h per day	58	104
>6 h per day	18	78
Study and/or work activities		
No use	23	9
<2 h per day	138	39
2–4 h per day	82	65
4–6 h per day	52	88
>6 h per day	34	124

**Table 3:** Means, standard deviation, F, and partial eta squared of boys’ and girls’ scores on CDPI, TAS-20, IAT, and BIAS.

Psychological Variables	Males	Females	F(1,399)	Partial Eta Squared	*p*
CDPI	32.54 (14.09)	38.69 (14.31)	15.66	0.038	<0.001
TAS-20 F1	14.66 (4.08)	14.26 (4.62)	0.66	0.002	0.415
TAS-20 F2	18.73 (6.75)	18.58 (6.78)	0.04	0.000	0.834
TAS-20 F3	18.88 (4.14)	17.11 (4.42)	13.88	0.034	<0.001
TAS-20 Tot	52.26 (12.38)	49.95 (12.97)	2.73	0.007	0.099
IAT	26.95 (13.86)	22.39 (12.23)	10.75	0.026	0.001
BIAS	12.12 (5.92)	12.24 (5.52)	0.032	0.000	0.857

Note. CDPI = COVID Peritraumatic Distress Index; TAS-20 F1= Factor 1 of Toronto Alexithymia Scale-20; TAS-20 F2 = Factor 2 of Toronto Alexithymia Scale-20; TAS F3 = Factor 3 of Toronto Alexithymia Scale-20; TAS-20 Tot = Total score of Toronto Alexithymia Scale-20; IAT = Internet Addiction Test; BIAS = Bergen Instagram Addiction Scale.

**Table 4:** Means, standard deviation, F, and partial eta squared of boys’ and girls’ scores on CDPI, TAS-20, IAT, and BIAS.

Psychological Variables	No Distress (*n* = 106)	Mild to Moderate Distress (*n* = 223)	Severe Distress (*n* = 71)	F (2,396)	Partial Eta Squared	*p*
TAS-20 F1	12.20 (4.53) ^a^	14.91 (4.24) ^b^	15.96 (3.91) ^b^	22.34	0.101	<0.001
TAS-20 F2	14.62 (6.24) ^a^	18.60 (5.84) ^b^	24.68 (5.67) ^c^	64.69	0.246	<0.001
TAS-20 F3	16.81 (4.53) ^a^	17.82 (4.22) ^b^	18.31 (4.68) ^b^	5.11	0.025	0.006
TAS-20 Tot	43.62 (12.84) ^a^	51.34 (11.63) ^b^	58.94 (10.76) ^c^	42.08	0.175	<0.001
IAT	19.24 (12.34) ^a^	23.52 (11.58) ^b^	31.27 (14.35) ^c^	26.78	0.119	<0.001
BIAS	9.81 (4.22) ^a^	12.14 (5.07) ^b^	15.97 (7.04) ^c^	29.79	0.131	<0.001

Note. TAS-20 F1= Factor 1 of Toronto Alexithymia Scale-20; TAS-20 F2 = Factor 2 of Toronto Alexithymia Scale-20; TAS F3 = Factor 3 of Toronto Alexithymia Scale-20; TAS-20 Tot = Total score of Toronto Alexithymia Scale-20; IAT = Internet Addiction Test; BIAS = Bergen Instagram Addiction Scale. ^a,b,c^ = Means in rows, not sharing a common letter, differ significantly (*p* < 0.05).

**Table 5:** Pearson’s correlation analyses between emerging adults’ scores on TAS-20, CDPI, IAT, and BIAS.

Psychological Variables	TAS-20 Tot	CDPI	IAT	BIAS
TAS-20 Tot	1	0.45 **	0.43 **	0.35 **
CDPI	0.45 **	1	0.35 **	0.40 **
IAT	0.43 **	0.35 **	1	0.54 **
BIAS	0.35 **	0.40 **	0.54 **	1

Note. TAS-20 Tot = TAS-20 Total score; CDPI = COVID Peritraumatic Distress Index; IAT = Internet Addiction Test; BIAS = Bergen Instagram Addiction Scale. ** *p* < 0.001.

## Data Availability

The data presented in this study are openly available in FigShare at doi:10.6084/m9.figshare.16709275.
